# Assessment of two novel methods for detecting non-cavitated, smooth surface enamel lesions: an in vitro study

**DOI:** 10.1186/s12903-025-06181-1

**Published:** 2025-05-29

**Authors:** Joel M. White, Pragati Nahar, Puja Kukreti, Jonathan Mangum, Ann Solterbeck, Rachel Jensen, Leslie Plack, Ram Vaderhobli, Larry Jenson

**Affiliations:** 1 Department of Preventive and Restorative Dental Sciences, UCSF School of Dentistry, 707 Parnassus Avenue, San Francisco, CA 94105 USA; 2 UCSF School of Dentistry, 707 Parnassus Avenue, San Francisco, CA 94105 USA; 3 Incisive Technologies Pty Ltd, Level 6, 41 Exhibition Street, Melbourne, VIC 3000 Australia; 4 Statistical Revelations Pty Ltd., 41 The Parade Ocean Grove, Melbourne, VIC 3226 Australia; 5 Centre for Biopharmaceutical Excellence, Incisive Technologies, Level 11, 655 Elizabeth St, Melbourne, 3000 Australia

**Keywords:** Carious detection, Demineralization, ICDAS, Minimally Invasive Dentistry, Non-cavitated caries lesions

## Abstract

**Background:**

The objective of this study was to compare two new non-cavitated caries lesion detector methods, LumiCare (LC) and BlueCheck (BC), to each other and to the traditional visual/tactile method (TM) of non-cavitated enamel caries lesion detection on smooth surface lesions.

**Methods:**

In this non-inferiority study, forty extracted human teeth with International Caries Detection and Assessment System (ICDAS) level 1 and 2 smooth-surface lesions were evaluated in standard operatory conditions by three ICDAS-trained dentists using the traditional visual and tactile method, the LumiCare method, and the BlueCheck method of detection. For each endpoint of Sensitivity (Se) and Specificity (Sp), a generalized mixed model was fitted with positive (negative) agreement (yes/no) as the outcome and method (LC or BC), evaluator and evaluator *method included as fixed effects.

**Results:**

Statistical analysis shows that the LumiCare and BlueCheck methods of detection are substantially equivalent and compare favorably to the traditional method of lesion detection. Inter-rater reliability and intra-rater reliability ratings were moderate to good.

**Conclusions:**

This study supports the indications for use for both LumiCare and BlueCheck as aids to visualization of early carious lesions. LumiCare and BlueCheck are substantially equivalent to each other in their ability to differentiate between demineralized enamel and sound enamel. Each may help to educate patients, monitor lesion progression following re-mineralizing therapy, and train beginning dentists in the use of ICDAS.

**Supplementary Information:**

The online version contains supplementary material available at 10.1186/s12903-025-06181-1.

## Background

Non-cavitated caries lesion detection is an essential part of modern caries management strategies. It is well established that the progression of non-cavitated lesions can be reliably arrested if not reversed with remineralization interventions such as brushing with fluoride toothpaste, fluoride varnish, tricalcium phosphate, casein phosphopeptide-amorphous calcium phosphate, fluorides, self-assembling peptides, and synthetic nano-hydroxyapatite. It is also well-established that these therapies have the best chance of success when applied early in the demineralization process [1–5]. Additionally, it has been shown that deeper lesions require increased levels of re-mineralizing agents [6]. The detection of non-cavitated lesions also enables the clinician to obtain a more accurate caries risk assessment of a patient; non-cavitated lesions are an indication that the combination of pathological and preventive factors for a given patient is “out of balance,” thus leading to patient recommendations to control those risk factors [7]. Moreover, the ability to assess any changes in the number and severity of non-cavitated enamel lesions over time allows the dentist to evaluate the success of caries control strategies; understanding the activity of a lesion (arrested, re-mineralizing, progressing) is essential feedback **f**or both patient and clinician. The challenge for researchers studying minimally invasive techniques has been to develop clinical tools that can reliably and non-destructively differentiate between healthy enamel, active carious lesions, inactive carious lesions, and areas of developmental hypomineralization.

The accurate detection of non-cavitated lesions has proven to be a challenge. Areas of active demineralization can be missed entirely if there are no color changes. They can also be mistaken for arrested/inactive lesions, extrinsic staining, or hypomineralizations caused by other processes. Moreover, it is well known that carious demineralization of enamel can occur even before visual cues of a lesion are clinically evident [8–10]. The traditional method (TM) of non-cavitated lesion detection, best characterized as direct visualization and tactileization under visible white light, is still the most commonly used and considered the gold standard for studies on clinical lesion detection [11–14]. And yet, the evidence establishing the effectiveness of this method is less than conclusive**.** Systematic reviews on the accuracy of the traditional method provide a variety of conclusions, ranging from poor to adequate success in detecting non-cavitated lesions. What is clear from the systematic reviews is that no other method of detection has been found to be superior to the traditional method [11, 14, 15]. It should be noted that studies that focus on detecting smooth surface lesions are few, adding to the uncertainty in the accuracy of the traditional method. In addition to accuracy concerns, the traditional method’s reliance on the use of a dental explorer is problematic. It is now generally agreed that probing a demineralized enamel surface can lead to cavitation [16, 17]. This has led investigators to search for less potentially destructive methods of detection.

Several technologies have been developed in recent years to meet the need for improved and non-destructive detection methods. These include laser or light-induced fluorescence, fiber optic transillumination, optical coherence tomography, electrical impedance, bioluminescence, and others [11, 18–21]. While these technologies have proven to be useful, clinical adoption has been slowed by the cost of equipment required, clinical applicability, ease of use, and inconsistent sensitivity and specificity performance. One of the most recent methods introduced for non-cavitated enamel lesion detection is an oral rinse containing proprietary fluorescent starch nanoparticles that bind to porous, demineralized areas in enamel and then fluoresce when exposed to blue light utilizing a standard clinical composite curing lamp [22–25]. LumiCare (GreenMark Biomedical Inc.) has received FDA clearance to assist dentists in the visualization of caries lesions and has been shown to be clinically effective in distinguishing between the various clinical presentations of active lesions, inactive lesions, areas of hypomineralization, and healthy enamel. Another new method for non-cavitated enamel caries lesion detection is a solution that contains proteins having a specific affinity for porous hydroxyapatite. BlueCheck (Incisive Technologies Pty Ltd, Melbourne, Victoria, Australia) is an oral “paint” containing hemoglobin that has been minimally modified with a deep-blue colored dye that is applied directly to teeth for the detection of demineralized and hypo-mineralized dental tissues. The hemoglobin molecule shows an affinity for demineralized areas of enamel, and the blue dye makes the area of demineralization visible under normal white light in standard clinical environments.

The present study is a comparative investigation of these two new methods. The primary objective of this study was to assess the enamel lesion-detecting ability of LumiCare and BlueCheck (LC and BC) using the traditional visual/tactile method (TM) as a reference gold standard and to demonstrate that BC was non-inferior to LC. This was done through the comparison of the sensitivity (Se) and specificity (Sp) of each method using TM as the reference/gold standard method. Secondary objectives included the estimation of positive predictive value (PPV) and negative predictive value (NPV) for each method (LC and BC and a comparison between the two) and to assess the inter-rater agreement and intra-rater reliability (test–retest reliability) for all three methods (TM, LC, and BC). Though it is an invitro study, it investigates these methods on human teeth with naturally produced enamel lesions in a clinical setting. The hypothesis tested was that BC was equivalent to LC, as compared to the gold standard traditional method using a non-inferior study design.

## Methods

### Study design

This in vitro study is a comparison of two methods of enamel lesion detection. It is a non-inferiority design to establish substantial equivalency between the two methods. Human Ethics and Consent to Participate declarations: not applicable. Ethical approval was not required as the study was not human subjects research and therefore not FDA-regulated. Institutional Review Board approval was not required for using de-identified tooth specimens in an in vitro study. The de-identified tooth samples used in this study were collected for other purposes and donated to the University of California, San Francisco School of Dentistry. Investigators Self-Certification Form for Determining Whether Human Subjects are involved in Research When Obtaining Coded Private Information (data) and/or Biological Specimens, i.e.de-identified donated teeth, was completed following Human Research Protection Program Institutional Review Board guidance from UCSF, Federal-wide Assurance number FWA00000068, IORG Registration number IORG0000135. This study adhered to the Helsinki Declaration.

### Sample size calculation

The sample size was pragmatic based on the availability of appropriate teeth. The power of the study was checked by simulation. One thousand simulated datasets were created, assuming 30 teeth and, on average, 3 grids/tooth with positive results by TM and, on average, 7 grids/tooth with negative results by TM. It was assumed that sensitivity was the same for BC and LC at 77% and for specificity at 95%, and a non-inferiority margin for BC–LC was set at -10%. The results from the simulation showed the power for sensitivity to be > 80% and for specificity to be > 90%. Based on the power estimates, sample size calculations determined that 30 teeth would be sufficient. We included 40 teeth to account for any loss of specimens during the course of the study.

### Specimens

**Fig. 1:**
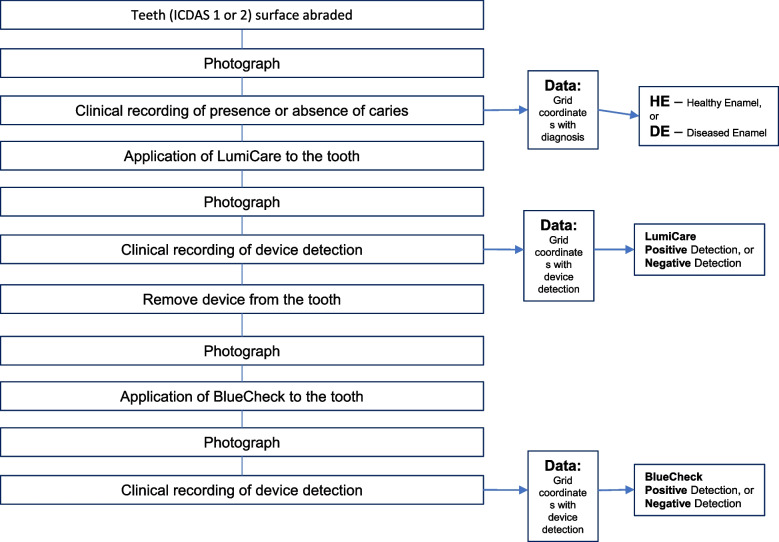
Experimental sequence

Forty extracted posterior human teeth that had identifiable smooth surface, non-cavitated lesions as determined by the International Caries Detection and Assessment System (ICDAS lesions 1 and 2) were used for the comparisons. The first detection method evaluated was the traditional method (TM) of visually and tactilely examining the surface of the tooth under white light to ascertain changes in the enamel that suggest carious demineralization. Cleaning and then drying of the tooth surface with compressed air was part of this process. As this method is generally considered the standard detection method, it was used as a gold standard in this study. The second method added LumiCare (LC) to the evaluation process, and the third method added BlueCheck (BC) to the evaluation process. Figure 1 shows the experimental sequence and data collection workflow.

### Tooth preparation

The forty specimens were selected from a biobank of teeth by an experienced dentist, trained in ICDAS. All lesions on the smooth surface of the teeth met the ICDAS criteria for initial lesions 1 and 2: visible demineralization indicated by the loss of normal gloss upon drying and/or visible enamel color changes of white, yellow, or brown, without cavitation. Following classification and selection, one surface of each tooth was selected, including the initial enamel lesion with healthy surrounding enamel, where the entire surface was cleaned and then lightly abraded with a fine diamond bur in order to remove any superficial, impermeable, remineralized layers in lesions that were arrested. The intent of the study was not to study lesion activity but only the ability of methods to differentiate healthy enamel and diseased (demineralized due to caries). Abrading the surface ensured that all lesions evaluated were “active,” i.e., lacking a remineralized layer**.** Abrading the surface also slightly reduced the curvature of the tooth surface, facilitating the entire process.

**Fig. 2:**
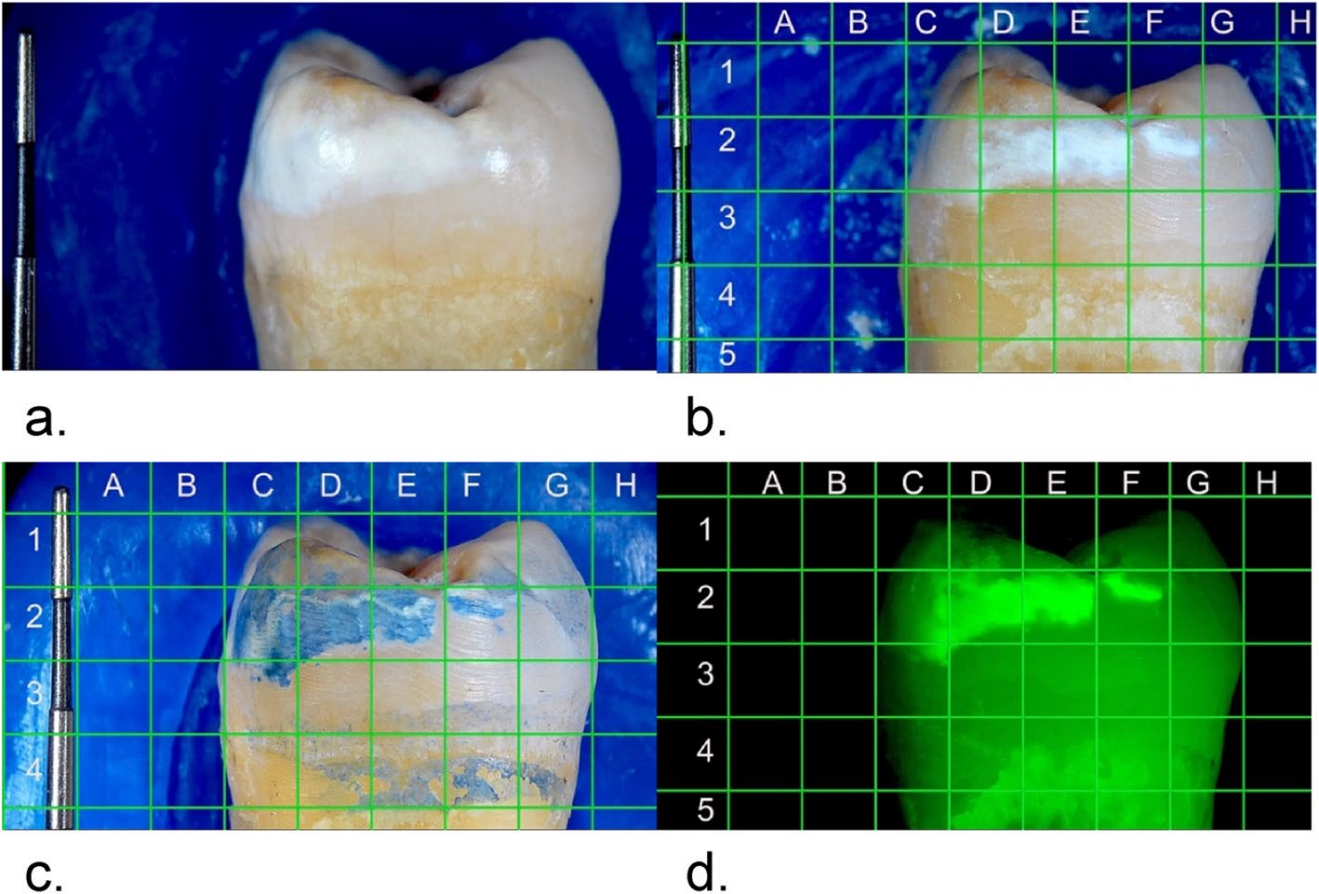
**a** is an example of an untreated specimen photographed in white light, (**b**) is the same specimen following abrasion with overlaid grid photographed in white light; (**c**) is the same specimen treated with BlueCheck and photographed under blue light; and (**d**) is the same specimen treated with LumiCare and photographed under white light

Following the abrasion process, each specimen was rinsed and dried, then mounted in a rigid wax mold and photographed at a repeatable distance from an intraoral camera and using white light. The resulting images were overlaid with a standardized, 2 mm × 2 mm positional grid pattern demarcating areas (samples**)** to be examined. These initial images became the standard for comparisons. The specimens were treated with the LC according to the manufacturer’s instructions and then returned to the original rigid mold and photographed again, but under blue light from an LED curing light (Dentsply SmartLite, iQ2) and a fluorescence filter set (for FITC Fluorescein). The resultant images were then overlaid with the standardized grid and became the comparison images for LC. Each specimen was then cleaned with water and dried. BC (Batch X2211082-02) was applied to each specimen as per the manufacturer’s instructions and then returned to the original rigid wax mold and photographed under white light. The resultant images were then overlaid with the standardized grid and became the comparison images for BC. Figure 2 shows an example of the three comparison images for one specimen.

Although a fully randomized cross-over study would have randomized the order of application (LC and BC), this approach was not followed as the prior application of BC hindered the emission of the wavelength of LC, thus affecting the results of the LC evaluation. However, prior application of LC did not influence the binding of BC and thus did not affect the results of the BC evaluation.

### Evaluation process

Three experienced dental clinicians, trained in ICDAS, were asked to be evaluators for the study, which was conducted in a standard clinical setting with available operatory lights and a computer screen for displaying the comparison images. Evaluators were provided with specimens, a dental mirror, a dental explorer, periodontal probes, loupes, and compressed air syringes. They were allowed to handle and manipulate the specimens as they viewed the comparison images on the computer screen. Evaluators were then asked to evaluate each of the standardized 2 mm x 2 mm grid areas (samples) and determine if the grid area had evidence of demineralized enamel indicating disease. The grid area was scored as “all or nothing” with 0 being no disease evident and 1 being any indication of disease evident. Signs that indicated demineralization/disease were defined by ICDAS 1–2, color (white), and surface texture (matte appearance). Grid areas that were unclear, as assessed by any one evaluator, were not included in the study. Evaluators were allowed to corroborate their image evaluations by examining the actual tooth specimen corresponding to the images. All evaluators evaluated all 40 specimens for each round of the study: TM, then LC, and then BC. All 40 specimens were in the same group. Each was evaluated by three different methods within that one group; a standard grid was placed on the photograph of the tooth. On average, there were 16 grids/tooth (min = 10, max = 20). For positive agreement, there were on average 6 grids/tooth identified by TM (min = 1, max = 12). For specificity, there were on average 10 grids/tooth assessed as negative by TM (min = 4, max = 16). A technician prepared each round of evaluations by randomizing the order of the specimens and then applying the LC or BC. Following each round, a retest of the evaluators was conducted using a randomly selected subset of four teeth (10% of the samples) to establish intra-examiner reliability. Examination results were recorded at the site onto specific data sheets for each specimen. Data were then entered into a standard database spreadsheet and imported into SAS V9.4 for statistical analysis.

### Statistical methods

This study employed a non-inferiority design.

#### Primary endpoints

For each tooth assessment, the presence/absence of disease for each grid segment was recorded for each evaluator for each method (TM, LC, BC). Positive agreement (sensitivity (Se)) between LC and TM and separately between BC and TM was determined for all grid segments where TM noted the presence of disease. Negative agreement (specificity (Sp)) between LC/BC and TM was determined for all grid segments where TM noted the absence of disease. The grid segment within a tooth represents a matched set (as it was assessed for TM, LC, and BC). For each endpoint (Se and Sp), a generalized mixed model was fitted with positive (negative) agreement (yes/no) as the outcome and method (LC or BC), evaluator, and evaluator *method included as fixed effects. For statistical analysis, tooth was included to account for clustering in the data, and the grid segment within the tooth was included to represent the matched set.

#### Secondary endpoints

PPV and NPV were analyzed as described for the primary endpoints of Se and Sp. From the model, the predicted probabilities of agreement (positive and separately negative) for each method were obtained, and the difference in agreement between BlueCheck and LumiCare (with 95% confidence limits) was obtained. To conclude substantive equivalence, a non-inferiority framework was employed, using a non-inferiority margin of 10%. If the lower 95% confidence limit for the difference in performance (sensitivity/specificity for BC-LC) was >—10%, then non-inferiority was concluded. Note: in this setup, if the lower 95% confidence limit for the difference in performance is > 0, this is consistent with the conclusion that BlueCheck demonstrated better performance than LumiCare.

#### Inter-evaluator agreement

The Interclass Correlation Coefficient (ICC) for agreement between evaluators was estimated separately for each method using a mixed model, where the result was the outcome (Se or Sp), and tooth*grid and evaluator were random terms. Using the estimated covariance parameters, the ICC was calculated as variance due to tooth ID*grid/total variance.

#### Intra-evaluator agreement: Test–retest reliability

Each evaluator repeated the assessment for all three methods: Traditional (TM), BlueCheck (BC), and LumiCare (LC) for a random selection (10% = 4 teeth) of teeth. The kappa statistic was determined for each method overall and by evaluator and reported with 95% confidence limits.

## Results

### Descriptive statistics

Forty teeth were examined. On average, there were 16 grid segments per tooth (maximum was 20). The total number of grids evaluated was 1,920 (Table 1).

**Table 1:** Overview of the data used in the study

Total Teeth	40	
Average Number of Grids per Tooth	16	
Total Grids Evaluated (× 3 Evaluators)	1920	
Total Grids, across 3 Evaluators, recorded as positive	TM positive	969
	LC positive	751
	BC positive	766

### Primary endpoint

**Fig. 3:**
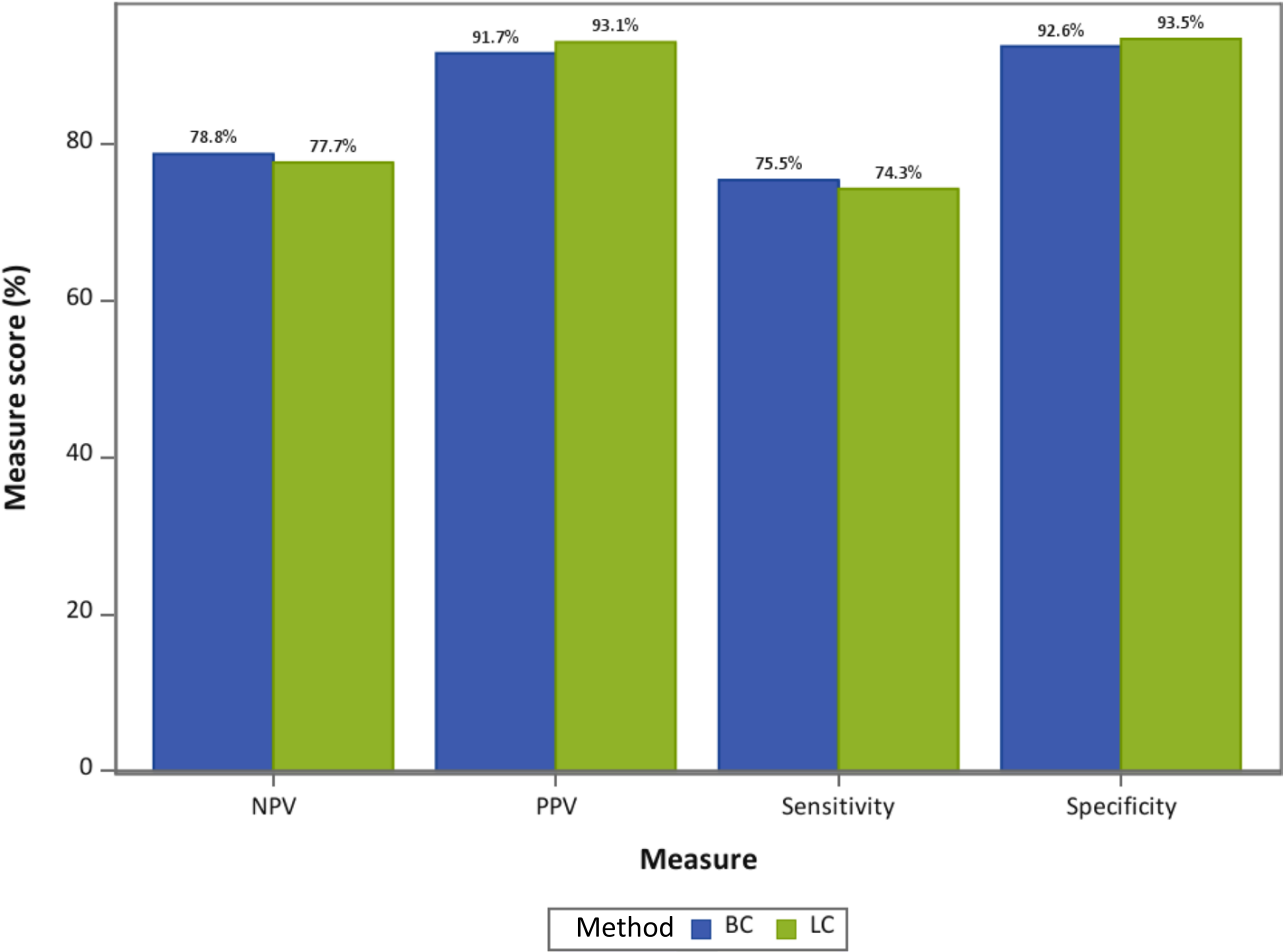
Performance by method (BC and LC)

Performance of LC and BC using TM as the gold standard. Figure 3 shows the overall performance of LC and BC for sensitivity and specificity (primary endpoints) as well as NPV and PPV (secondary endpoints) using TM as the reference (gold) standard. Sensitivity was 75.5% (CL 68.5%, 81.3%) for BlueCheck and 74.3% (CL 67.6%, 80.1%) for LumiCare. Specificity was 92.6% (CL 88.2, 95.4%) for BlueCheck and 93.5% (CL 89.3%, 96.1%) for LumiCare. PPV was 91.7% (CL 87.9%, 94.4%) for BlueCheck and 93.1% (CL 89.5%, 95.5%) for LumiCare. NPV was 78.8% (CL 71.9%, 84.4%) for BlueCheck and 77.7% (CL 71.2%, 83.2.%) for LumiCare.

### Secondary endpoint: performance comparisons between methods

**Fig. 4:**
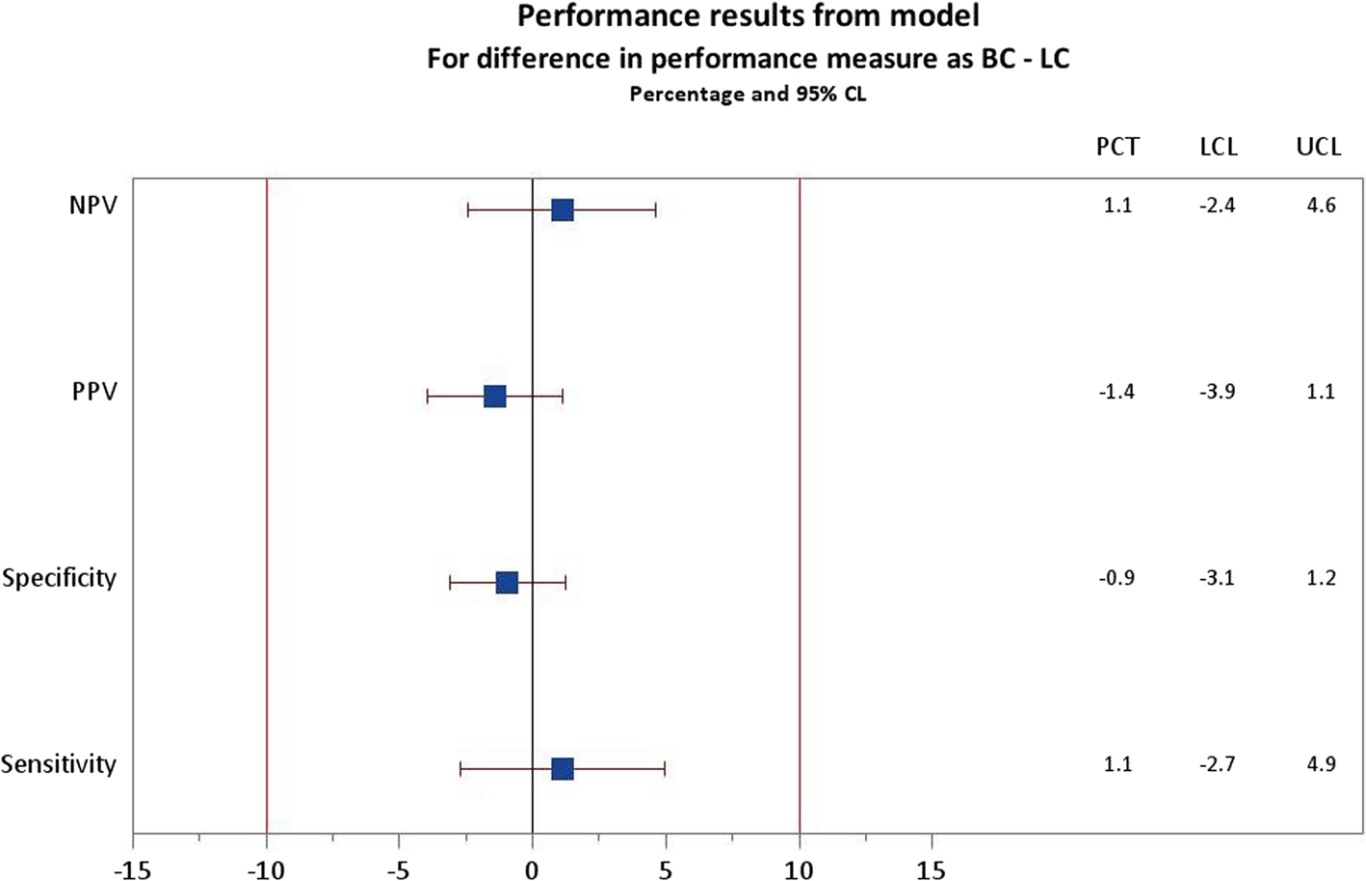
Performance results between Lumicare (LC) and BlueCheck (BC)

The assessment of non-inferiority of BC compared with LC is shown in Fig. 4 for the primary endpoints of Se and Sp and for the secondary performance indicators of PPV and NPV. Figure 4 shows that BlueCheck is substantially equivalent (non-inferior) to LumiCare. For both performance indicators, the lower 95% confidence limit for BC-LC is above the pre-defined margin of -10%**.**

### Reliability

#### Inter-rater reliability

Inter-rater agreement was assessed using the ICC. Results (by method) are presented in Table 2.

**Table 2:** ICC by method

Method	ICC
BlueCheck	0.78
LumiCare	0.69
Traditional	0.54

#### Intra-rater reliability

A simple kappa statistic was obtained for the test–retest agreement for each method and evaluator and is presented in Tables 3 and 4. These results show that reviewer RV was the most consistent and LP the least. For both JMW and LP, the assessment using visual-tactile inspection (traditional method) was the least consistent. All three examiners achieved almost perfect test–retest reliability using LumiCare and BlueCheck according to established guidelines [26]. For TM, two out of three evaluators (JW and RV) showed almost perfect agreement, with one (LP) showing substantial agreement.

**Table 3:** Test-re-test reliability assessed by the kappa statistic

Method	Observed agreement	Expected agreement by chance	Kappa (95% confidence limits)
BlueCheck	93.85%	50.36%	0.88 (0.81, 0.94)
LumiCare	92.31%	52.04%	0.84 (0.76, 0.92)
Traditional	86.15%	49.63%	0.73 (0.63, 0.82)

**Table 4:** Test re-test reliability by method and by reviewer

Reviewer	Method	Observed agreement	Expected agreement by chance	Kappa (95% confidence limits)
JMW	BlueCheck	96.88%	54.83%	0.93 (0.84, 1.00)
JMW	LumiCare	93.75%	55.86%	0.86 (0.72, 0.99)
JMW	Traditional	89.06%	54.39%	0.76 (0.59, 0.93)
RV	BlueCheck	98.44%	50.29%	0.97 (0.91, 1.00)
RV	LumiCare	100.00%	50.78%	1.00 (1.00, 1.00)
RV	Traditional	100.00%	50.44%	1.00 (1.00, 1.00)
LP	BlueCheck	86.57%	50.03%	0.73 (0.57, 0.89)
LP	LumiCare	83.58%	50.84%	0.67 (0.49, 0.84)
LP	Traditional	70.15%	48.50%	0.42 (0.23, 0.61)

## Discussion

As the modern caries lesion management paradigm has become more established, it is increasingly apparent that the ability to differentiate between healthy enamel and non-cavitated lesions is of the utmost importance. The traditional visual and tactile method currently used to detect non-cavitated enamel lesions lacks good support for efficacy, reliability, and usability [27]. It is clear that newer, non-destructive methods of detection are needed to augment the traditional method, thus enhancing the clinician’s ultimate diagnostic accuracy and subsequent therapeutic plan. This study has provided a good opportunity to evaluate two novel detection methods for non-cavitated, smooth surface lesions. The study supports the conclusion that both detection methods, BC and LC, compare favorably to the reference standard (TM) in their ability to detect non-cavitated enamel lesions. The study also shows a favorable comparison *between* the two new methods for detecting non-cavitated, smooth surface enamel lesions supporting the hypothesis. BC and LC were found to be substantially equivalent in their ability to detect non-cavitated lesions. BlueCheck was substantially equivalent to LumiCare in the primary endpoints of sensitivity and specificity, as well as the secondary endpoints of positive predictive value and negative predictive value.

As for the reliability of the two methods, the results shown in Table 2 demonstrate substantial agreement between evaluators for LC and BC and moderate agreement for TM. Values between 0.5 and 0.75 are considered moderate and above 0.75 are good according to the guidelines from Landis and Koch 1977 [26]. BlueCheck demonstrated the highest level of agreement. The results shown in Tables 3 and 4 demonstrate almost perfect test–retest reliability. The inter- and intra-rater reliability results may suggest that these methods can improve the ability of a dentist who is less trained in ICDAS to more closely approximate expert ICDAS evaluation. This would suggest that they could have a role in training dental students in the use of ICDAS.

This study supports the indications-for-use for both methods; both have been shown to be good aids to visualization. It is an interesting finding from this study that the least consistent method was the gold standard traditional method. This suggests that the new tools show better consistency and an important improvement over the traditional method of visualization/tactile. It should be said that, like other caries lesion detection dyes, assisting visualization is not necessarily equivalent to improving or enhancing the accuracy of the dentist. This study did not specifically measure any additional benefit that either product brings to the accuracy of detection using ICDAS. This would require a different study that measures all three methods against some other gold standard so that the traditional method could be compared with and without the other two methods. While this study shows that LumiCare and BlueCheck perform well as measured against the traditional method, the experimental method did not test the use of either one to enhance the accuracy of the traditional method. However, one might reasonably conclude that assisted visualization would ultimately result in better detection accuracy.

### Limitations of the study

A limitation of this study is that it is an in vitro study; clinical application may show different results, though LumiCare has been shown to be effective in an in vivo study [22]. Plans for a future clinical study for BlueCheck are underway. While both detection methods show promise for all types of caries lesions, this study was limited to only active, smooth surface lesions on permanent teeth with no cavitation. Similarly, only active lesions were studied; no attempt at differentiating between active, inactive, and developmental lesions was made; in fact, the study was designed to eliminate inactive lesions from evaluation. Also, no attempt was made to detect pre-visible lesions. The use of ICDAS as a gold standard is limited. While these two detection methods performed well when compared to ICDAS, this study did not compare these methods or the ICDAS method to a more substantiated gold standard such as histological analysis.

The clinical implications of this study are that both LumiCare and BlueCheck can be used to help *confirm* the initial clinical judgment of the dentist. And because they both assist visualization, they can also be easily used as educational aids to help patients better understand their caries status and the ongoing progress of caries treatment modalities. This study should be viewed in the context of the MID and caries management by risk assessment (CAMBRA) paradigms. Unlike the case with cavitated lesions, non-cavitated lesions are not identified to pursue surgical intervention but to establish whether the patient’s oral conditions are “in balance.” The existence of even one area of non-cavitated demineralization is information that the patient is likely “out of balance” and that appropriate therapeutic interventions are indicated. In that case, the need for absolute accuracy in identifying all active lesions is not as critical; the patient is either in balance or not, with little significance given by the number or severity of non-cavitated lesions.

Although the available comparative literature on non-cavitated lesion detection includes an extensive list of novel methodologies, few are directly comparable to this study in that they either focus on tooth surfaces other than smooth surfaces or use a reference standard other than the traditional visual/tactile method. It should be noted that the majority of comparative studies focusing on non-cavitated detection concern occlusal and approximal lesions. In fact, one of the most recent systematic reviews on non-cavitated detection contains only two studies that include smooth surface lesions [11]. Another recent systematic review shows no studies that include smooth surface lesions [15]. It should also be noted that many comparable studies in enamel lesion detection fail to report the full set of Se, Sp, inter- and intra-rater reliabilities, PPV and NPV as this study does [28]. It has been suggested that these four metrics should always be reported together so that an appropriate conclusion may be made about a diagnostic test [29].

Each method has unique characteristics that may prove to be advantageous in their clinical application. The BlueCheck method requires less specialized equipment than the LumiCare method, which requires the use of a specific curing light and viewing glasses. Though both products dissipate rapidly and are easily removed, the LumiCare is not visible in white light, so patients may find it less objectionable. The BlueCheck method can be more easily targeted to specific areas on specific teeth than the LumiCare method, which involves the entire mouth. Both LumiCare and BlueCheck can be used to help confirm the dentist’s initial clinical judgments, better determine caries risk assessment, monitor the progress of remineralization efforts, and improve patient education. Visual enhancement indicators may also prove to be an aid in educating beginning practitioners in the use of the ICDAS for caries lesion identification.

## Conclusion

The results of this study support the conclusion that the BlueCheck caries lesion detector is substantially equivalent to the LumiCare caries lesion detector in detecting non-cavitated, smooth surface enamel caries lesions in extracted human teeth. Both methods compare favorably with the traditional method of visualization and tactileization under white light and show moderate to good inter-rater and intra-rater reliability. This study also supports the indications-for-use for both methods; both have been shown to be aids to visualization.

## Supplementary Information

Supplementary Material 1.

## Data Availability

All data generated or analyzed during this study are included in this article and its supplementary material files. Further enquiries can be directed to the corresponding author.

## Abbreviations

ICDAS: International Caries Detection and Assessment System
MID: Minimally invasive dentistry
TM: Traditional Method
LC: LumiCare
BC: BlueCheck
PPV: Positive predictive value
NPV: Negative predictive value
SAS: Statistical Analysis Software
ICC: Intercorrelation coefficient
CAMBRA: Caries management by risk assessment
